# Assessment of Knowledge and Attitudes of Traumatic Dental Injuries among Saudi Dental Students: A Multicenter Cross-Sectional Study

**DOI:** 10.1155/2020/8814123

**Published:** 2020-07-25

**Authors:** Sundus Bukhary

**Affiliations:** Division of Endodontics, Department of Restorative Dental Science, College of Dentistry, King Saud University, PO Box 45347, Riyadh 11512, Saudi Arabia

## Abstract

**Introduction:**

The aim of this multicenter cross-sectional study was to assess the knowledge and attitudes of senior dental students in Riyadh, Saudi Arabia, regarding the management of traumatic dental injuries.

**Materials and Methods:**

A stratified random sample of fourth- and fifth-year dental students in different dental schools was given a two-part questionnaire; the first part included demographic questions, while the second part included case scenario questions related to knowledge and attitudes regarding the emergency management of concussion, crown fractures, luxation injuries, root fracture, and alveolar bone fracture. Data were statistically analysed using chi-square and ordinal logistic regression tests. The significance was set at *p* < 0.05.

**Results:**

A total of 359 dental students participated in this study. The proportion of students from all dental schools with correct responses to each set of questions was as follows: alveolar bone fracture (78.4%), root fracture (70.1%), intrusive luxation (57.1%), complicated crown fracture (39.7%), uncomplicated crown fracture (35.5%), extrusive luxation (35%), concussion (31.1%), and lateral luxation (26.8%). The mean overall knowledge score of the participants was 3.421 ± 0.49. No statistically significant difference was found on any of the questions regarding the gender or year of study (*p* > 0.05).

**Conclusion:**

Based on the findings of this study, dental students' knowledge of the management of traumatic dental injuries in the Riyadh region of Saudi Arabia ranges from low to moderate. Thus, there is a need to improve the knowledge of management of traumatic dental injuries among dental students.

## 1. Introduction

Traumatic dental injuries (TDIs) are confronted as common public health problems sustained by children, adolescents, and adults [1, 2]. The world TDIs incidence rate has been reported to be 2.82 per 100 person/year, with 22.7% prevalence in primary dentition and 15.2% in permanent dentition, with the highest prevalence (18.1%) in 12-year-old children [3]. However, the incidence of TDIs in Saudi Arabia is higher than that reported in other countries [4, 5].

TDIs can range from simple chipping off of the enamel surface, crown fractures, root fractures, and luxation injuries to severe damage to supporting tooth structures. Significant damage to the developing permanent successor occurs when it is directly involved in the trauma causing developmental disturbances and malalignments that requires a multidisciplinary team of skilled specialists to support the conservative treatment in a growing child [6, 7]. Thus, early diagnosis and close monitoring after severe TDIs are essential to prevent aesthetic, functional, and psychological problems in young children [8]. To ensure favourable healing of traumatized dental tissues, it is imperative that the patient be provided with quick emergency intervention and treatment at critical times of the healing phase. Guidelines for TDIs treatment were released by the International Association of Dental Traumatology (IADT), the American Association of Endodontists (AAE), and the American Academy of Pediatric Dentistry (AAPD) to provide a professional reference with periodic updates to be followed by the dental practitioner to ensure as efficient treatment approaches as possible [9–11].

The dental student is expected to be competent prior to graduation in identifying and managing traumatic dental emergencies in primary and permanent dentitions and referring severe cases to a specialized dental-care provider. Hence, it is critical to assess the existing knowledge of dental traumatology among dental students as they play a key role in providing the necessary intervention in the near future. There are many studies conducted in several countries that showed that dental practitioners lack the adequate knowledge of emergency treatment of TDIs [12–15]. However, to our knowledge, studies on the assessment of dental students' knowledge and attitude toward the managements of TDIs are lacking. Therefore, the purpose of this study was to assess the knowledge and attitudes of senior dental students studying in different dental schools in the Riyadh region of Saudi Arabia regarding the management of traumatic dental injuries including crown fractures, root and alveolar bone fractures, and luxation injuries.

## 2. Materials and Methods

A multicenter cross-sectional, questionnaire-based study was conducted among dental students at different dental schools in Riyadh, Saudi Arabia, over a period of three months (October–December 2019). The inclusion criteria were undergraduate students (males/females) in their fourth and fifth years of dental training. The dental schools included in the study were from King Saud University (KSU), King Saud bin Abdulaziz University for Health Sciences (KSAU), Riyadh Elm University (REU), Al-Farabi Colleges, and Dar al Uloom University (DAU).

The study was approved by the Institutional Review Board of King Saud University in Riyadh, Saudi Arabia (E-19-3921), and prior permission was procured from the schools' authorities for conducting the study. A self-administered questionnaire was developed and tested among a random sample of dental students to ensure the validity and practicability of responses. The questionnaire items were based on the International Association of Dental Traumatology guidelines [9].

A stratified random sampling technique was used to collect a sample of 359 participants, representing 26.2% of the dental students from all schools included in the study (total of 1370 students). Enrolment numbers of participants were retrieved from the student directory of each of the universities, and every consecutive fourth- and fifth-year dental student was selected to participate in the study. All the participants voluntarily gave their informed consent, and upon completion of the questionnaire, the responses were kept anonymous.

A self-designed, close-ended, online questionnaire was designed for data collection which included questions on demographic characteristics (gender, year, and school they attended) and their knowledge and attitudes regarding traumatic dental injuries. The questions were presented in a case scenario format, and the participants were instructed to choose the single best answer (Table 1).

The collected data were entered into spreadsheet software (Microsoft Excel 16.0). Descriptive statistics was used to obtain the frequency distribution of the data. Inferential analysis was performed using the Statistical Package for Social Sciences (SPSS, IBM Version 22.0). Contingency tables and chi-square tests (*χ*2) were used to determine the correlations between correct responses to the knowledge questions and participant demographic characteristics. The overall knowledge score as an ordinal outcome was calculated by scoring one point for each correct answer and zero points for wrong answers, with a maximum possible score of eight points, and ordinal regression analysis was performed to measure the effect of gender and the year of study. The level of significance was set at 0.05.

## 3. Results

A total of 359 students participated in this study of which 193 were fourth-year students and 166 were fifth-year students. There were 211 (58.77%) female and 148 (41.12%) male participants, respectively. The demographic characteristics of the respondents are shown in Table 2. The majority of the respondents answered that their knowledge about TDIs was inadequate (90.8%), and most of them wanted to attend an additional course on TDIs (91.64%).

The overall correct responses from students in all dental schools to each question were as follows: the majority of the students correctly answered the questions regarding alveolar bone fracture (78.4%) and root fracture (70.1%). More than half of the students correctly answered the question about intrusive luxation (57.1%). However, other questions were correctly answered by the following proportions of students: complicated crown fracture (39.7%), uncomplicated crown fracture (35.5%), extrusive luxation (35%), concussion (31.1%), and lateral luxation (26.8%). The mean overall knowledge score of the participants was 3.421 ± 0.49 out of 8.

Female participants scored more correct responses than male participants for all questions. However, the results did not depict any significant difference in the response between male and female participants except for question 3 (*p* < 0.05), which assesses the course of action for an intruded tooth that shows an open apex on the radiograph. With respect to the year of study, no statistically significant difference was found on any of the questions between fourth- and fifth-year students (*p* > 0.05) (Table 3).

Table 4 represents the effect of gender and the year of study on the overall knowledge score. Both gender and the year of study showed no significant difference in the overall knowledge score (*p* > 0.05).

## 4. Discussion

Traumatic dental injuries are an inevitable clinical situation that requires a thorough knowledge to support the correct diagnosis and treatment. To the best of the author's knowledge, this is the first study that assessed dental students' knowledge in key aspects of dental trauma management, ranging from concussion to crown and root fracture, luxation injuries, and alveolar bone fracture conducted in the city of Riyadh. In this study, the level of knowledge of dental students was found to be ranging from low to moderate, with an overall mean knowledge score of 3.421 ± 0.49. Similarly, many previous studies have reported the lack of knowledge about the management of dental trauma among dental practitioners [12–15]. The results also revealed that gender and the year of study had no significant effect on the mean knowledge score. However, Al-Shamiri et al. [16], reported that fifth-year dental students had significantly higher knowledge scores than the fourth-year students had, and this inconsistency could be because one dental school was included in his study compared to this multicenter cross-sectional study. Senior dental students were selected in the present study as questionnaire participants, as these students had already taken the didactic courses on dental trauma to enable us to assess their knowledge regarding the management of such trauma.

Crown fractures are common in anterior teeth, with an incidence of 18–22% of all TDIs, 22–44% of which are uncomplicated fractures and 11–15% of which are complicated crown fractures [17]. The main focus in managing a case of uncomplicated crown fracture is the restoration of the aesthetics and function of the affected tooth, which can be achieved by various adhesive restorative techniques [9]. In the present study, only 35.5% of the students responded correctly to the question on uncomplicated crown fracture. When asked about complicated crown fracture with an immature apex, 39.7% of the participants responded correctly. The results of our study are comparable to those studies that showed insufficient knowledge regarding the emergency management of crown fractures [18, 19].

When the knowledge of dental students about the emergency management of luxation injuries was assessed, more than half of the participants (59.6%) knew that the correct intervention for the intrusion of a tooth with an immature apex was to allow the tooth to erupt naturally without intervention and, if there is no change in the position after a few weeks, to start orthodontic or surgical repositioning of the affected tooth [9]. However, their knowledge regarding the management of extrusive luxation did not seem to be satisfactory, as only 35% of participants knew the correct management, and only 26.7% of participants knew the correct management of lateral luxation injury. Such findings coincide with those of the study of Al-Haj Ali et al., who reported inadequate knowledge of general practitioners and dental specialists regarding the management of luxation injuries [14].

The current study showed satisfactory results regarding the students' knowledge of root fracture management, as 70% of students gave the correct response. In regard to the management of alveolar bone fracture, the majority of the students (78.4%) answered correctly. These results are in accordance with those of a previous study that showed a satisfactory knowledge about root and alveolar bone fractures among dentists [18].

It has been reported that the retention of knowledge about TDIs decreases with time [20, 21]. Different educational interventions were reported to improve the dental trauma knowledge among dental students. Repeated lectures were found to be effective at improving the knowledge and retention of knowledge. The retention of knowledge concerning dental trauma was higher after reminder lectures than before the lecture in undergraduate students [22, 23]. AlZoubi et al. suggested that dental trauma education should be held as a mandatory course for the dental practitioner to take annually or biannually [23]. In this study, the vast majority of the participants (91.64%) wanted to attend an additional TDIs course, and most (90.8%) believed that they had inadequate TDIs knowledge. These results stressed the need for additional dental trauma educational materials to be given to dental students before and after graduation.

The delayed treatment approach of TDIs might alter the facial appearance of children and negatively affect their daily life due to lack of social acceptance [24]. Low rates of treatment of dental trauma are observed worldwide, and this could be attributed to the fact that tooth fractures and luxation injuries are not perceived as a clinical situation which needs immediate treatment [25, 26]. Rodd et al. showed that dental students reported that the perceived level of confidence in managing dentoalveolar trauma was the least compared to various procedures [27]. Thus, dental students should have the perception toward the treatment of TDIs as expensive, time consuming, and involving multidisciplinary approaches with long follow-up visits.

Some limitations of this study should be acknowledged; the study is not representative of all dental students in Saudi Arabia as only the Riyadh Province was included. Also, the study was limited in its scope and was not designed to assess the curricula or teaching methods of the dental schools. Future research could be undertaken to ascertain the differences in the teaching methods and curricula of dental schools in Saudi Arabia regarding the courses of dental trauma.

## 5. Conclusions

Within the limitations of this study, we conclude that dental students' knowledge regarding the management of traumatic dental injuries in the Riyadh region of Saudi Arabia ranges from low to moderate. The present findings provided baseline information on the existing knowledge and highlight the need to improve the knowledge of dental students regarding TDIs and their management by adding additional courses covering dental trauma in dental undergraduate programs.

## Figures and Tables

**Table 1:** The questionnaire.

Q	Situation	Correct answer
1	If a case of uncomplicated crown fracture (without pulp exposure) of the central incisor with a mature apex came to your clinic, the immediate treatment would be	If a tooth fragment is available, it can be bonded to the tooth; otherwise, cover the exposed dentin with glass ionomer or composite dressing

2	If there is a case of root fracture that is near the cervical area of the tooth with a mature apex, the immediate treatment would be	Reposition the displaced coronal segment of the tooth as soon as possible, stabilize the tooth with a flexible splint up to 4 months, and follow-up if pulp necrosis develops, and root canal treatment of the coronal tooth segment to the fracture line is indicated to preserve the tooth

3	If a case with an intruded tooth that shows an open apex on the radiograph came to your clinic, the treatment would be	Allow eruption without intervention, and if no movement within few weeks, initiate orthodontic or surgical repositioning

4	If a case with extrusive luxation of a mature tooth came to your clinic, the immediate treatment would be	Immediate repositioning and splinting for 2 weeks

5	If a case with enamel-dentin-pulp fracture of the central incisor with an immature apex came to your clinic and the trauma occurred 3 hours ago, the immediate treatment would be	Preserve pulp vitality by pulp capping or partial pulpotomy

6	If a case with alveolar bone fracture in a 17-year-old boy, who fell off his bike two hours ago, with a mobile fractured segment and several teeth moving together that causes malocclusion came to your clinic, how would you manage?	Reposition any displaced segment, suture gingival laceration if present, and stabilize the segment for 4 weeks

7	A 26-year-old patient came to your clinic complaining of a tender tooth while touching or tapping due to blow from a kid's toy. Upon examination, the tooth has no increased mobility and sensibility tests give normal results and you diagnose the case with concussion. How would you manage?	No treatment is needed; just monitor the pulpal condition for at least one year

8	A 45-year-old patient received a blow to his face from a basketball that caused his front tooth to be displaced lingually. The tooth is immobile, and upon percussion, it gives a metallic sound and negative response to sensibility tests. You diagnosed the case with lateral luxation. How would you manage?	Reposition the tooth to disengage it from its bony lock and gently reposition it into its original location. Stabilize the tooth for 4 weeks and monitor the pulpal condition

**Table 2:** Demographic distribution of participants with respect to the dental school, gender, level of their TDIs knowledge, and their desire to attend a TDIs course.

	School *N* (%)	Gender *N* (%)	Your knowledge about TDIs is *N* (%)	Do you want to attend an additional course of TDIs? *N* (%)
Fourth-year students	KSU = 72 (37.30%) KSAU = 30 (15.54%) REU = 59 (30.56%) Al-Farabi = 28 (14.50%) DAU = 4 (2.07%)	Male: 57 (29.54%) Female: 136 (70.46%)	Adequate: 14 (7.26%) Inadequate: 179 (92.74%)	Yes: 176 (91.19%) No: 17 (8.80%)

Fifth-year students	KSU = 44 (26.50%) KSAU = 46 (27.71%) REU = 18 (10.84%) Al-Farabi = 47 (24.35%) DAU = 11 (6.62%)	Male: 91 (54.81%) Female: 75 (45.19%)	Adequate: 19 (11.44%) Inadequate: 147 (88.55%)	Yes: 153 (92.16%) No: 13 (7.83%)

Total	KSU = 116 (32.31%) KSAU = 76 (21.16%) REU = 77 (21.44%) Al-Farabi = 75 (20.89%) DAU = 15 (4.17%)	Male: 148 (41.12%) Female: 211 (58.77%)	Adequate: 33 (9.19%) Inadequate: 326 (90.80%)	Yes: 329 (91.64%) No: 30 (8.35%)

**Table 3:** Frequency distributions of dental students' responses to the questions with association to gender and the year of study.

Gender							
	Female		Male		OR	95% CI	
	*N*	%	*N*	%			

Q1	81	36.8	54	33.8	0.874	0.570	1.340
Q2	154	70.0	103	64.4	0.774	0.502	1.195
Q3	139	63.2	78	48.8	0.554^*∗*^	0.367	0.838
Q4	73	33.2	60	37.5	1.208	0.789	1.849
Q5	81	37.0	63	39.6	1.118	0.735	1.701
Q6	90	40.9	59	36.9	0.844	0.555	1.283
Q7	70	31.8	48	30.0	0.918	0.591	1.428
Q8	62	28.2	40	25.0	0.849	0.535	1.350

Year of study							
	Fourth year		Fifth year		OR	95% CI	
	*N*	%	*N*	%			

Q1	68	34.9	62	36.5	1.072	0.698	1.647
Q2	128	65.6	125	73.5	1.454	0.926	2.283
Q3	117	60.0	97	57.1	0.886	0.583	1.345
Q4	72	36.9	55	32.4	0.817	0.530	1.260
Q5	76	39.2	63	37.1	0.914	0.598	1.397
Q6	82	42.1	60	35.3	0.752	0.492	1.149
Q7	56	28.7	61	35.9	1.389	0.894	2.159
Q8	49	25.1	51	30.0	1.277	0.806	2.024

**Table 4:** Ordinal regression analysis to assess the effect of gender and the year of study on the overall score of knowledge.

Variables	Knowledge score (mean ± SD)	OR	95% CI		*p*
*Gender*	**3.421** **±** **0.49**				
Female	3.41 ± 1.63	1			
Male	3.15 ± 1.64	1.23	0.860	1.770	0.253

*Year of study*	**3.465** ± **0.50**				
4th year	3.32 ± 1.64	0.95	0.660	1.370	0.782
5th year	3.37 ± 1.59	1			

## Data Availability

The data used to support the findings of this study are available from the corresponding author upon request.
